# Environmentally exploitable biocide/fluorescent metal marker carbon quantum dots†

**DOI:** 10.1039/d0ra06383e

**Published:** 2020-11-26

**Authors:** Hanan B. Ahmed, Hossam E. Emam

**Affiliations:** Chemistry Department, Faculty of Science, Helwan University Ain-Helwan Cairo 11795 Egypt hananbasiony@gmail.com +201097411189; Department of Pretreatment and Finishing of Cellulosic Based Textiles, Textile Industries Research Division, National Research Centre, Scopus Affiliation ID 60014618 33 EL Buhouth St., Dokki Giza 12622 Egypt hossamelemam@yahoo.com +201008002487

## Abstract

Carbon quantum dots are currently investigated to act as safe/potent alternatives for metal-based nanostructures to play the role of probes for environmental applications owing to their low toxicity, low cost, chemical inertness, biocompatibility and outstanding optical properties. The synthesis of biocide/fluorescent metal marker carbon quantum dots with hydrophilic character was performed *via* a quite simple and green technique. The natural biopolymer that was used in this study for the synthesis of carbon quantum dots is fragmented under strong alkaline conditions. Afterwards, under hydrothermal conditions, re-polymerization, aromatization and subsequent oxidation, the carbonic nanostructures were grown and clustered. Dialysis of the so-produced carbonic nanostructures was carried out to obtain highly purified/mono-dispersed carbon quantum dots with a size distribution of 1.5–6.5 nm. The fluorescence intensity of the synthesized carbon quantum dots under hydrothermal conditions for 3 h was affected by dialysis, however, the fluorescence intensity was significantly increased *ca.* 20 times. The synthesized carbon quantum dots were exploited as fluorescent markers in the detection of Zn^2+^ and Hg^2+^. The prepared carbon quantum dots also exhibited excellent antimicrobial potency against *Bacillus cereus*, *Escherichia coli* and *Candida albicans*. The detected minimal inhibitory concentration for the dialyzed CQDs towards the tested pathogens was 350–450 μL mL^−1^. The presented approach is a simple and green technique for the scaled-up synthesis of biocide/fluorescent marker carbon quantum dots instead of metal-based nanostructures for environmental applications, without using toxic chemicals or organic solvents.

## Abstract 2

Synthesis of biocide/fluorescent metal marker carbon quantum dots with hydrophilic character for the detection of Zn^2+^ and Hg^2+^.

## Graphical abstract

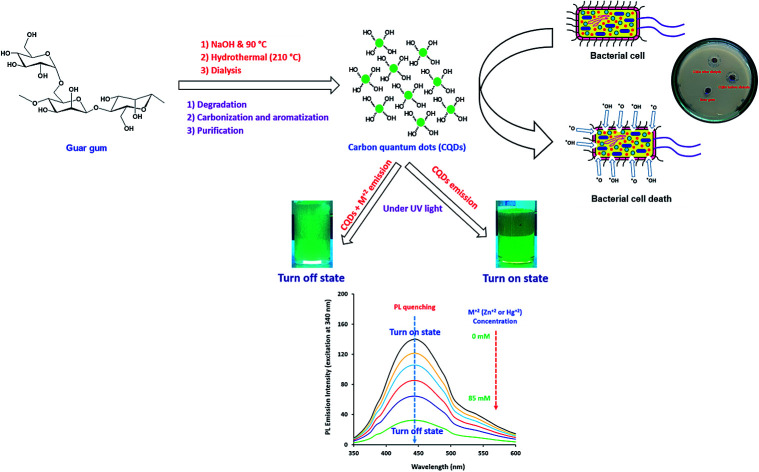

## Introduction

Carbon quantum dots (CQDs) have emerged to act as safe/potent reagents for environmental applications owing to their low toxicity, low cost, chemical inertness, good biocompatibility and outstanding optical properties. CQDs have recently been investigated and defined as a new member in the family of carbon nanostructures. CQDs exhibit a discrete quasi-spherical geometric shape and particle sizes below 10 nm, which are reported to possess more intriguing characters and activities compared with other carbon nanomaterials.^1^ This has consequently positioned carbon quantum dots as potential alternatives to the other members of the carbon nanostructures family.^2–4^ It was reported that Xu *et al.* first investigated CQDs as fluorescent carbonaceous nanostructures during the electrophoretic purification of single walled carbon nanotubes.^5^ Afterwards, numerous studies were extensively concerned with investigating various methods for the synthesis of CQDs and studying their outstanding and superior properties, such as chemical inertness, amazing luminescence, low cost, excellent biocompatibility, and abundance of raw materials.^6–12^

CQDs are mainly composed of C, H and O elements, while different organic and inorganic starting materials can be successively exploited in their synthesis. Polysaccharides as natural organic polymers can be successively employed in the generation of CQDs, which are supposed to be decorated with different functional groups. Exploiting polysaccharides in the nucleation of CQDs is known to result in the existence and decoration of oxygen-containing moieties for superior water solubility and gives the opportunity for further functionalization with different species.^13^ To control the geometric shape and particle size, a variety of synthetic strategies were reported in order to adopt simple, cost-effective, and large scale approaches. Tuning of the experimental conditions such as temperature and reaction duration was also reported for significant control over the geometric shape and size of the as-produced CQDs. All of the recently reported synthetic routes are generally classified into two main approaches: the traditional top-down approach such as chemical oxidation^14–16^ and electrochemical synthesis^17,18^ and the bottom-up approach like microwave assisted synthesis^19,20^ and the hydrothermal approach.^21–23^

Zhang *et al.* performed a one-step hydrothermal method for the synthesis of CQDs from l-ascorbic acid and the as-prepared CQDs with a diameter of 2.0 nm were found to exhibit a relatively high photoluminescence (PL) efficiency.^24^ Yang *et al.* reported another study for the synthesis of amino-functionalized fluorescent CQDs *via* a hydrothermal method by the carbonization of chitosan at a mild temperature of 180 °C.^23^ The hydrothermal technique is described as an efficient and direct method, where it mainly takes place *via* two steps: polymerization then carbonization reactions. This approach could be widely employable for the synthesis of CQDs due to the low energy consumption, environmentally safe nature and feasibility of controlling the reaction conditions.^25^ Various purification techniques such as centrifugation, dialysis, filtration, electrophoresis, silica column chromatography and high performance liquid chromatography (HPLC) were carried out after the successive synthesis of CQDs to obtain mono-disperse/highly purified CQDs.

In the last decade, numerous studies have reported the preparation of various types of nanostructures to be superior in applications with different purposes such as biomedicine and catalysis.^26–36^ Fluorescent carbon quantum dots (CQDs) are a fascinating class of nanostructures compared to metallic nanoparticles in various purposes due to their low toxicity, biodegradability and biocompatibility.^1,37–40^ Therefore, they are considered as superior alternatives for metal-based nanomaterials. CQDs are reported to be widely applicable in various fields such as drug delivery, photodynamic therapy, photo-catalysis, electro-catalysis, bio-sensing, chemical sensing, bio-imaging and optoelectronic devices.^41,42^

A wide range of antimicrobial agents as antiseptics and disinfectants are used for the inactivation of microorganisms in order to prevent hazardous infections,^43–46^ however, these antimicrobial agents are disadvantageous due to their toxicity and extreme irritation, resulting in more dangerous health problems such as mucous membrane irritation and contact dermatitis. Additionally, some microbial species adapt and become resistant against such reagents over time.^47^ Therefore, the investigation of CQDs as alternative antimicrobial reagents with higher potency and less toxicity is urgently required and extensively pursued.

On the other hand, CQDs are often employed as good candidates for metal detection due to their biocompatibility. Some heavy metals like zinc or iron are essential for body functions and are rarely harmful in their adjustable concentration where they have a vital role in metabolism. However, some other heavy metals are very harmful for humans even in trace amounts, like mercury, lead and cadmium.^48,49^ These toxic metals could be easily agglomerated in the body and coordinatively bonded to enzymes and nucleic acids to corrupt their normal biological functions.^49,50^

Guar gum as a galactomannan polymer is a natural/biodegradable, non-ionic and water soluble polysaccharide that is extracted from the endosperm of cluster bean seeds.^51^ Guar gum is mainly constructed of α-(1,4)-linked β-d-manno-pyranose backbone branched with α-d-galactose(1,6-linked-α-d-galacto-pyranose).^52^ Guar gum is extensively applied in different fields because of its superior ability to alter the viscosity of aqueous solution, rheological characters and thickening.^53^ The hydroxyl groups as the main functional groups are responsible for the reactivity and functionalization of guar gum macromolecules.^54^ Numerous studies reported the exploitation of guar gum functional groups and their effects on the physical and chemical characteristics of guar gum in the blending, grafting and manufacturing of various composites with natural and synthetic polymers.^54,55^

Herein, a facile and green approach is successively investigated for the nucleation of hydrophilic CQDs using guar gum. The prepared CQDs were exploitable as probes for environmental applications to act as antimicrobial reagents and fluorescent metal markers. The synthesis was performed under hydrothermal conditions with sodium hydroxide as a strong alkali base. Dialysis was performed to obtain mono-dispersed and highly purified CQDs. The effectiveness of the presented methodology in the synthesis of CQDs was affirmed *via* several instrumental analyses like UV-visible spectroscopy, Zetasizing, transmission electron microscopy, FT-IR, Raman spectroscopy and NMR spectroscopy. Consequently, the fluorescence properties of the synthesized CQDs were examined and the superior fluorescence sensitivity of the as-prepared CQDs was exploited for the detection of zinc(ii) and mercury(ii) ions. The quenching mechanism of the fluorescence was also studied. The antimicrobial potency of the as-synthesized CQDs was estimated *via* the Kirby-Bauer disk diffusion method with evaluation of the minimal inhibitory concentration.

## Experimental sections

### Materials and chemicals

Guar gum was supplied from El-Nasser Company for Pharmaceuticals and Chemicals – Egypt. Sodium hydroxide (NaOH, 99%), copper nitrate (Cu(NO_3_)_2_, 99%), lead chloride (PbCl_2_, 98%), ferric nitrate (Fe(NO_3_)_3_, 98%), zinc acetate (Zn(CH_3_COO)_2_, 99%) and mercury acetate (Hg(CH_3_COO)_2_, 99%) were supplied by Merck, Darmstadt-Germany. Potassium bromide (KBr, 99%), potassium iodide (KI, 99%), magnesium chloride (MgCl_2_, 98%), cadmium nitrate (Cd(NO_3_)_2_, 99%), ferrous sulfate (FeSO_4_, 99%) and nickel chloride (NiCl_2_, 97%) were purchased from s.d. Fine-Chem Limited, Mumbai – India. All materials and chemicals were used as received and aqueous solutions were prepared with deionized water.

### Synthesis of hydrophilic CQDs

First, 40 g L^−1^ of NaOH was used to dissolve 10 g L^−1^ guar gum and the reaction solution was kept under magnetic stirring for two hours at 90 °C. The color of the solution gradually changed from transparent colorless to yellowish. Afterwards, the reaction solution was transferred to a vertical hydrothermal autoclave reactor and then placed in the oven for complete nucleation of CQDs at 210 °C for different durations (3, 6 and 12 h). Lastly, the reaction solution with a dark brown color was left in open air to be cooled then dialyzed with distilled water by using pur-A-lyzer dialysis kits (MW_CO_ 6–8 kDa from Sigma-Aldrich) to obtain uniform-sized/purified CQDs for further analyses. The dialysis process is a purification method used to separate excess unreacted reactants or byproducts from the as-required nanostructures in the colloidal sample solution since the excess of such undesirable byproducts could result in coagulation and agglomeration of the suspended CQDs in the prepared sample to non-favorable enlarged structures. So, dialysis as a purification technique was carried out directly after preparation of the samples in distilled water, where an apparatus called a dialyzer was used. Here, a bag with a specified membrane containing the colloidal solution of the sample was suspended in a vessel through which fresh distilled water flows continuously for 24 hours, and consequently, the undesirable products could diffuse through the membrane of the dialysis bag leaving behind the preserved as-required nanostructures.

### Instrumental analyses and characterization

Absorption spectra for guar gum and the synthesized CQDs were collected in the wavelength range of 250–750 nm using a UV-visible spectrophotometer (Cary 100 UV-Vis, UV–Vis-NIR Systems, from Agilent). Topographical features and size distributions of the prepared CQDs were estimated with a High Resolution Transmission Electron Microscope from Japan (JEOL-JEM-1200). The diameters of CQDs were evaluated by 4 pi analysis software (from USA) for 50 particles at least. The mean size and poly-dispersity for the synthesized CQDs were measured by using a Zetasizer analyzer (Malvern Nano ZS, from Malvern Instruments Ltd – UK) *via* dynamic light scattering at 25 °C in an insulated chamber. Raman spectra for the synthesized CQDs before and after dialysis were obtained by using an alpha300R – confocal Raman imaging (WITec from focus innovations – Germany). The spectral measurement was analyzed with diffraction-limited spatial resolution in the range of 500–3500 cm^−1^. Infrared spectral analysis was performed by using a Jasco FT/IR 6100 spectrometer. The absorbance spectra were detected in the range of 500–4000 cm^−1^ using 15 points for smoothing, 4 cm^−1^ resolution, and 64 scans with a scanning rate of 2 mm s^−1^. The nuclear magnetic resonance (^1^H-NMR and ^13^C-NMR) spectra were recorded on a Jeol-Ex-300 NMR spectrometer (JEOL – Japan). Photoluminescence spectra for the synthesized CQDs in the ultraviolet-visible spectral range were detected by a spectrofluorometer (JASCO FP8300). The measurements were carried out at room temperature with excitation at 340 nm while emission was detected.

### Fluorescent detection of Zn^2+^ and Hg^2+^

The prepared CQDs were evaluated for their fluorescence sensitivity to be exploited in the detection of heavy metals as fluorescent metal markers. A series of metal ions including Mg^+^, Zn^2+^, Cu^2+^, Fe^2+^, Fe^3+^, Cd^2+^, Pb^2+^, Hg^+^, Ni^+^, Br^−^, and I^−^ were prepared with concentrations of 200 mM. The utility of the as-prepared CQDs as fluorescent metal markers was carried out at room temperature in PBS buffer (pH 7.0). First, 0.5 mL of CQDs was added to 85 mM of metal salts in PBS buffer solution. The solutions were thoroughly mixed, and after 60 min the fluorescence emission spectra were measured at room temperature specifically, CQDs were applied in the detection of Zn^2+^ and Hg^2+^ at different concentrations ranging from 4–85 mM. The fluorescent detection was performed by using a spectrofluorometer (JASCO FP8300) with excitation at 340 nm.

### Antimicrobial potency

The antimicrobial potency of the as-synthesized CQDs against different pathogenic species was estimated *via* the qualitative method of a Kirby-Bauer disk diffusion technique.^45,56^ In this test, three different pathogenic species of +ve gram bacteria species (*Bacillus cereus*), −ve gram bacteria species (*Escherichia coli*) and fungal species (*Candida albicans*) were tested. To evaluate the lowest concentration of the as-prepared CQDs, which showed an observable inhibition in microbial growth, the minimal inhibitory concentration (MIC) from CQDs solution was detected. In the disk diffusion test (inhibition zone technique), different tested bacterial strains were grown in media to prepare pathogenic suspensions. Then, 100 μL of bacterial suspension was spread onto agar plates corresponding to the broth in which it was maintained. A 10 μL aliquot of non-dialyzed or dialyzed CQDs was placed on the middle of the plates and then incubated at 37 °C for 24 h. The diameters of the inhibition zones were detected in millimeters using slipping calipers according to NCCLS, 1997.^57^ In MIC determination, serial dilutions from CQDs solutions (0–1000 μL mL^−1^) were prepared and then added to the plates. After incubation at 37 °C for 24 h, the colony forming units (CFU) were counted for each dilution.^44,57^

## Results and discussion

### Mechanism for synthesis of hydrophilic/fluorescent CQDs

**Fig. 1:**
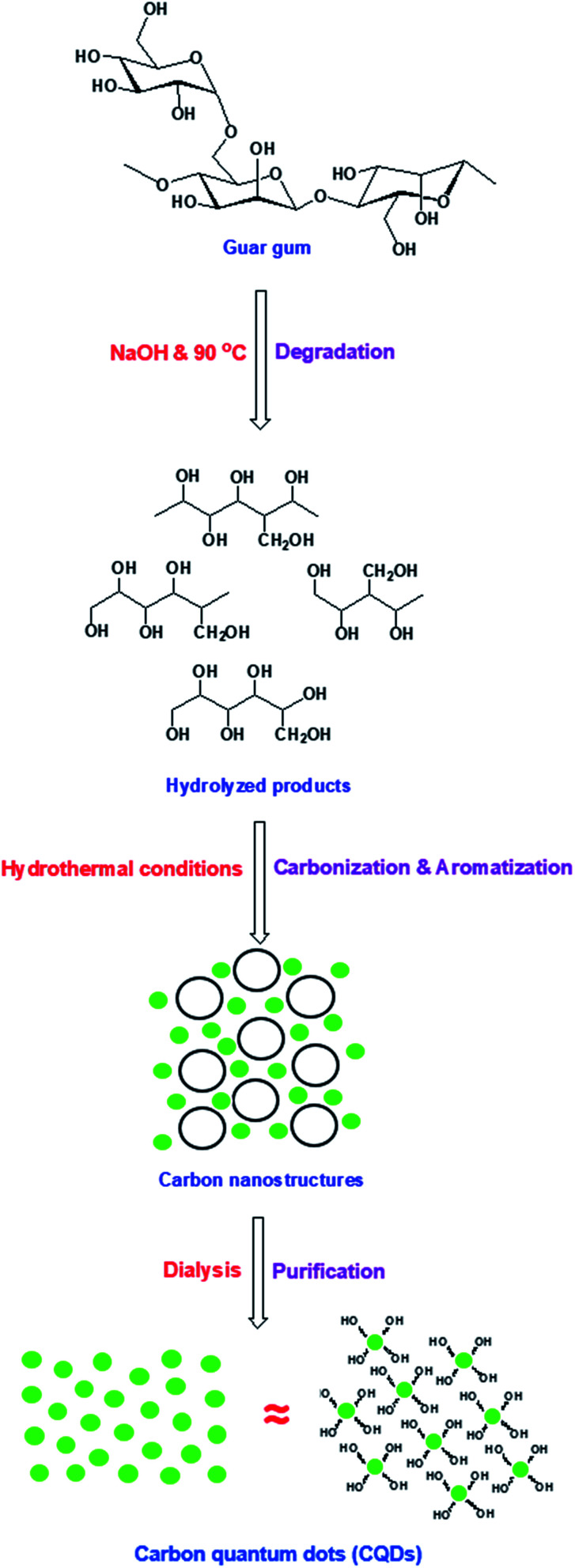
Suggested mechanism for synthesis of fluorescent CQDs from guar gum under hydrothermal conditions.

The fabrication of biocide/fluorescent metal marker CQDs was successively carried out in a one-step and facile technique under hydrothermal conditions from basic dissolved guar gum, as schematically presented in Fig. 1. According to literature, the mechanism for the synthesis of hydrophilic CQDs from alkali dissolved guar gum was postulated as follows: firstly, guar gum macromolecules are supposed to be hydrolyzed and fragmented under the effect of NaOH as a strong alkali base. Afterwards, under hydrothermal conditions, re-polymerization, aromatization and subsequent oxidation proceeded to give carbon nanostructures, which were consequently grown and clustered.^58^ Lastly, dialysis was performed on the sample colloidal solution in order to eliminate the unreacted molecules and any byproducts (crystalloids), which could drive the agglomeration of carbonic nanostructures (colloids), and consequently to obtain uniform-sized/purified CQDs with regular and stable geometric shapes.^58^ Guar gum was initially hydrolyzed into mannose and galactose, which were further fragmented into furfural intermediates,^25,59^ then consequently polymerized and aromatized to give CQDs as aromatic graphite sheets decorated with hydroxyl groups on their surfaces. In this process, the color of alkali hydrolyzed gum solution turned yellow then a reddish brown color.^60,61^

### Characterization of CQDs

**Fig. 2:**
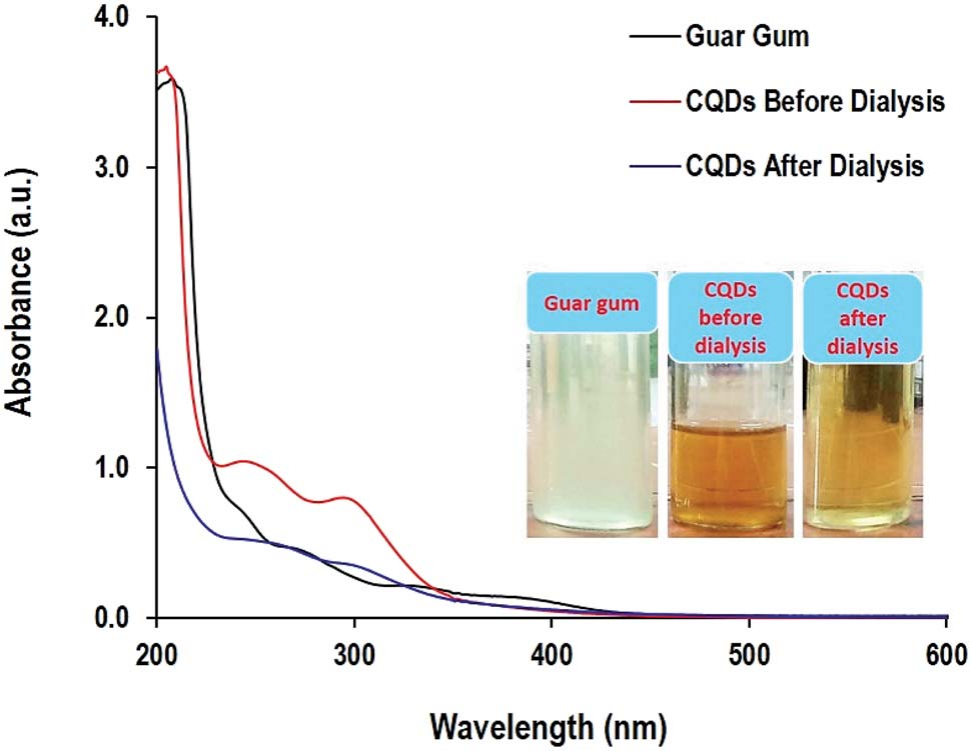
Absorbance spectra and the photographic image for the guar gum and the prepared CQDs after 12 h under hydrothermal conditions.

The optical properties of guar gum and CQDs before and after dialysis are presented in Fig. 2. The visual observation of the solutions showed that the turbid white color of guar gum turned dark yellow then reddish brown solution after the hydrothermal conditions, while after dialysis, it appeared to be a pale yellow. The colloidal solution of CQDs before dialysis showed two absorbance peaks that are typical for CQDs in the ultraviolet region at 250 nm and 300 nm, characteristic for a pi-electron (π–π*) C=C transition of the aromatic sp^2^ bond and n–π* transition of C=O functional groups, respectively.^62,63^ This data is in agreement with the literature,^64^ in which the optical absorption spectra of CQDs are typically in the ultraviolet range. However, after dialysis the intensity of the absorbance peaks was diminished and slightly shifted to longer wavelengths of 275 and 340 nm, which could be attributed to the purification of CQDs through the elimination of non-favorable unreacted molecules and byproducts *via* dialysis, which preserved the aromatized CQDs. Carbon quantum dots are characterized by spherical structures and are constructed from aromatic sheets of carbon atoms combined in highly organized nanostructures. Absorption spectra are mainly correlated to fluorescence, which results from the inter-construction of CQD building structures with heteroatoms.^65–71^

**Fig. 3:**
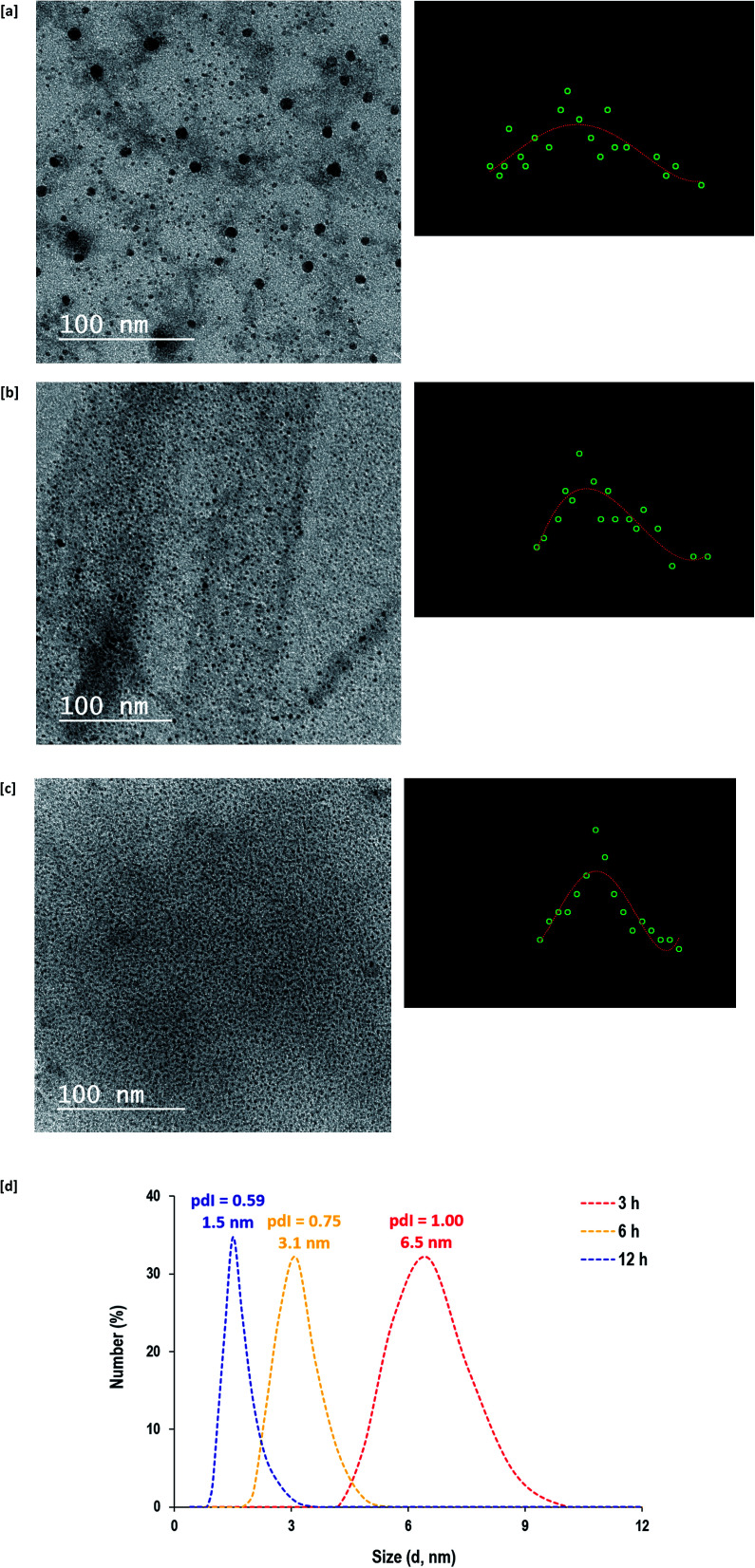
[a–c] Transmission electron micrographs for CQDs prepared under different hydrothermal times; [a] 3 h, [b] 6 h and [c] 12 h. [d] Zetasizer analyzer data.

The topographical features and geometric shape of the synthesized CQDs under hydrothermal conditions for 3 hours (Fig. 3a), 6 hours (Fig. 3b) and 12 hours (Fig. 3c) are shown in the transmission electron microscope (TEM) micrographs from which their size distributions were also estimated and plotted. Regardless of the hydrothermal duration, all of the as-prepared CQDs were spherical in shape with sizes smaller than 10 nm. By prolonging the hydrothermal reaction from 3 h to 12 h, smaller sizes and narrower size distributions of CQDs were produced. The particle size diameters (size distribution) of CQDs synthesized under hydrothermal conditions for 3 h, 6 h and 12 h were estimated to be 5.1 nm (1.7–10.2 nm), 2.2 nm (1.1–3.5 nm) and 1.7 nm (1.0–2.5 nm), respectively. Therefore, CQDs that were prepared with 12 h of hydrothermal conditions were shown to be homogeneous, well-dispersed and controllably clustered in the reaction medium with quite small sizes.

The particle size analyzer measurements for the prepared CQDs are presented in Fig. 3d. Even though the measurement of particle size was performed with a different technique (dynamic light scattering), similar particle sizes (6.5 nm for 3 h, 3.1 nm for 6 h and 1.5 nm for 12 h) were detected. It was also observed that the poly-dispersity index (pdI) of the obtained CQDs decreased from 1.00 to 0.75 and 0.59 due to longer hydrothermal duration from 3 h to 6 h and 12 h, respectively. This could be explained in that the longer hydrothermal duration provided more opportunities for re-polymerization and aromatization to generate controllable sizes of CQDs. Therefore, the zetasizer data further confirmed the TEM observations for the successive seeding of small sized and controllable CQDs.^33,43^ Generally, the analyzed data demonstrates the compatibility of alkali hydrolyzed guar gum in the generation of hydrophilic CQDs under hydrothermal conditions, however, performing the reaction for 12 hours was more preferable to generate well-dispersed/homogeneous and highly size-regulated CQDs.

**Fig. 4:**
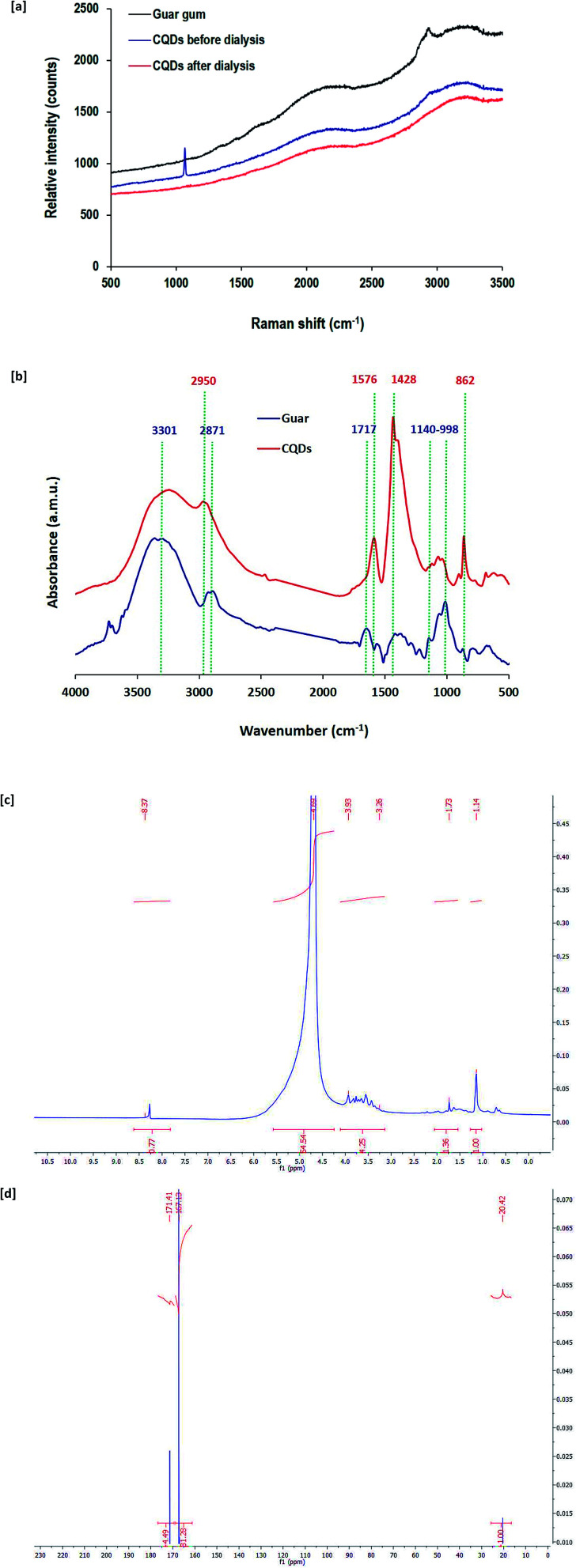
[a] Raman spectra, [b] FTIR spectra, [c] ^1^H NMR and [d] ^13^C NMR for CQDs prepared at 12 h hydrothermal time.

Raman scattering is a vibrational molecular spectroscopy that is valuable for the investigation of changes in the molecular structure of guar gum after its exploitation in the synthesis of CQDs. Fig. 4a represents the Raman spectral data for native guar gum and CQDs prepared by alkali hydrolyzed gum before and after dialysis. For native guar gum, three characteristic bands were detected for O–H, C–H and C=C at Raman shifts of 3237 cm^−1^, 2947 cm^−1^ and 2089 cm^−1^, respectively. After exploitation of gum in the nucleation of CQDs, a new band at 1068 cm^−1^ attributed to asymmetric C–O–C appeared and the characteristic band of C–H at 2947 cm^−1^ even after dialysis totally vanished. Moreover, after using guar gum in the synthesis of CQDs, the two bands of O–H and C=C became less intense. Additionally, the C–O–C band completely disappeared after dialysis. These findings are consistent with the removal of undesirable/unreacted molecules and byproducts after dialysis,^65–71^ to give only the purified small sized CQDs as highly crystalline graphite sheets with hydroxyl groups as surface decorating hydrophilic groups.

To verify the chemical structure of the synthesized CQDs, FT-IR analysis was also performed (Fig. 4b). FT-IR spectra are presented for both pristine guar gum as well as CQDs. Interpretation of the FT-IR data is presented in accordance to the literature.^72^ The pure gum spectrum exhibited a band typical for hydroxyl groups at 3301 cm^−1^ and the characteristic band for the aliphatic group was visible at 2871 cm^−1^. The spectral chart also exhibited a band typical for free carboxyl groups at 1717 cm^−1^. In addition, a band typical for glycosidic bonds between the monomeric units of gum macromolecules was observed at 998–1140 cm^−1^. FT-IR spectra for CQDs were different from the pure gum spectrum, which affirms the transformation of the gum macromolecular structure into CQDs. It could be observed that bands typical for gum coming from glycosidic bonds appeared to be less intense. Meanwhile, the characteristic band for the aliphatic group completely disappeared and at the same time, a new band corresponding to un-substituted C=C bonds appeared at 2950 cm^−1^. In addition, two bands that are typical for aromatic structures and C–H bending were detected at 1428 cm^−1^ and 862 cm^−1^, respectively. On the other hand, bands typical for carboxyl groups became sharper with shifts to 1576 cm^−1^, while that for hydroxyl groups still appeared after using gum in the nucleation of CQDs.

^1^H NMR and ^13^C NMR spectral data for CQDs nucleated from guar gum under hydrothermal conditions are also presented in Fig. 4c and d, respectively. ^1^H NMR spectral data revealed that characteristic bands were detected at 1.1 ppm and 1.7 ppm for sp^3^ C–H protons. The bands at 3.3–3.9 ppm and 4.7 ppm are typically assigned to protons of the hydroxyl decorative groups and protons attached to carbonyl groups, respectively. Moreover, a tiny band at 8.4 ppm corresponds to the aromatic or sp^2^ protons. The ^13^C NMR spectrum showed three characteristic bands for the prepared CQDs at 20.4, 176.1 and 171.4 ppm, which are assigned to C–H sp^3^ carbons, C=C sp^2^ aromatic carbons and C=O sp^2^ carbons, respectively.

So, all of the above data from UV-visible absorption spectra, Raman spectra, FT-IR, ^1^H NMR and ^13^C NMR indicated that the growth of CQDs from alkali hydrolyzed guar gum was successful under hydrothermal conditions through carbonization, cyclization and aromatization. This led to the nucleation of graphite sheets of CQDs with hydroxyl surface decorative groups, which are responsible for the hydrophilic potency of the as-nucleated CQDs.

### Fluorescence sensitivity of the as-synthesized CQDs

The fluorescence properties are reported to be related to the decorative surface groups rather than their size.^64^ Wu *et al.* showed that the fluorescence sensitivity of CQDs is mainly correlated to the special surface states that consist of decorative functional groups such as hydroxyl groups on the edge of the carbon backbone.^21^ The surface states are ascribed as fluorescence centers based on the synergetic hybridization of the carbon core and chemical groups.^24^ Therefore, the optical properties of the so-synthesized CQDs in the current approach were due to the decorative hydroxyl groups from alkali hydrolyzed guar gum macromolecules with good overlap of the π–π* and n–π* transitions for the aromatized nucleated CQDs. It must be mentioned that the surface decorative groups can slightly regulate the absorption location, leading to colorful CQDs and endowing them with the possibility for colorimetric detections.

**Fig. 5:**
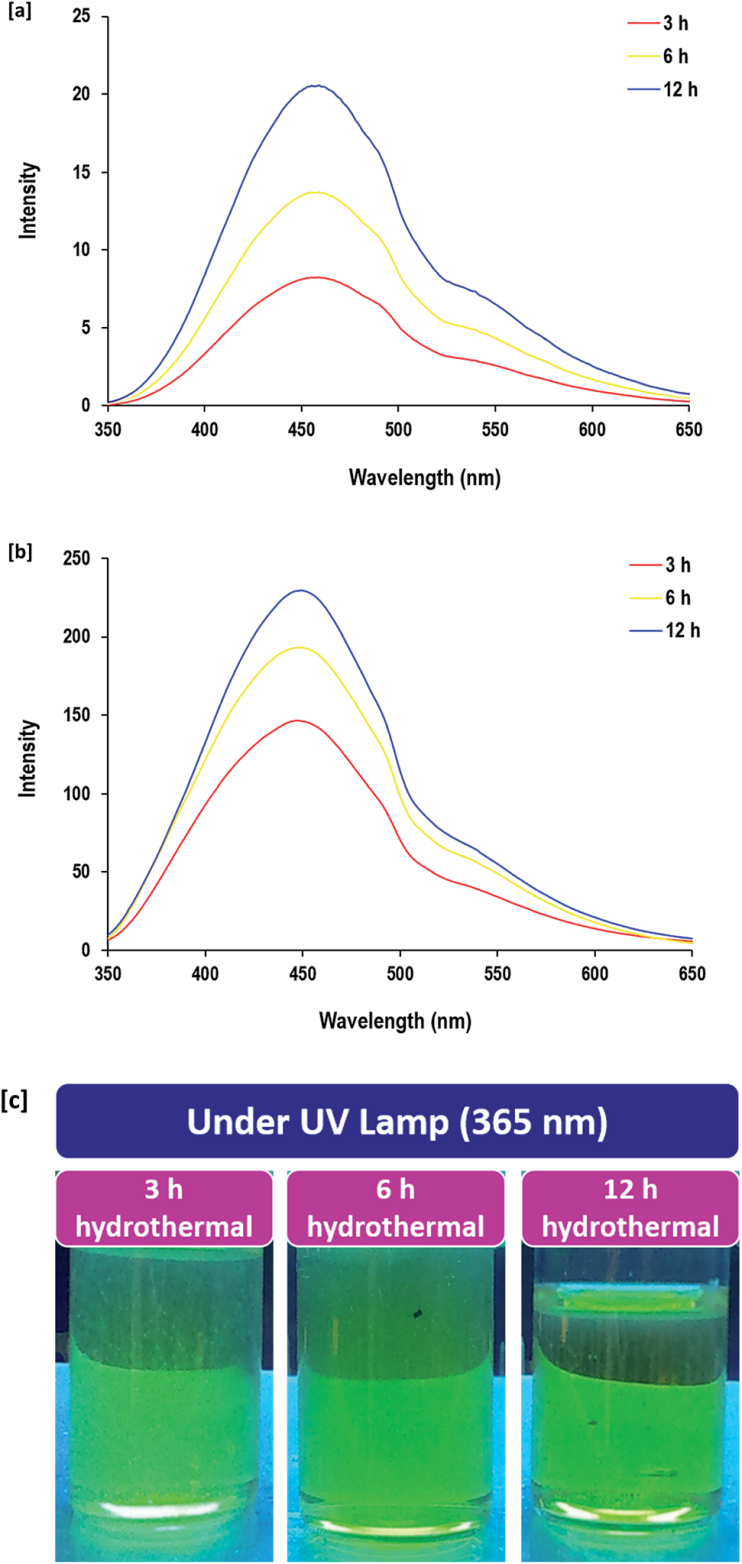
Fluorescence spectra (FL) for CQDs prepared under different hydrothermal times; [a] before dialysis and [b] after dialysis. [c] Photographic image for the CQDs after dialysis under UV lamp.

Therefore, the optical properties of the CQDs were investigated *via* FL spectroscopy and the FL emission spectra after excitation at 340 nm are shown in Fig. 5. The quantum yield of the as-synthesized CQDs that were prepared under hydrothermal conditions for 12 h and dialyzed was estimated to be 13.6% according to the reported method in the literature.^73^ The photographic images for the prepared samples under an ultraviolet lamp show that the synthesized CQDs emitted a green color in the UV region. The excited CQDs exhibited a FL emission band at 445 nm, which corresponded to the green region as reported in the literature.^74–77^ The intensities of FL emission were very low (*I* ≤ 21) before dialysis and were considerably increased by factor of 20 after dialysis under hydrothermal conditions for 3 h. The plotted spectra showed that prolonging the hydrothermal reaction duration from 3 h to 12 h was accompanied by a significant increase in the FL band intensity from 146 to 230, while it was increased by factor of 10 after dialysis, which was attributed to the removal of the undesirable/unreacted molecules and byproducts as non-fluorescent structures after dialysis. The phenomena of the green fluorescence for the as-synthesized CQDs was due to the existence of O–H as surface decorative groups.^78^ FL features of CQDs are known to be correlated to the size-induced quantum-confinement effect, therefore, a longer reaction duration allows for the nucleation of more size-regulated CQDs with superior FL sensitivity.

### Fluorescence detection of Zn^2+^ and Hg^2+^

**Fig. 6:**
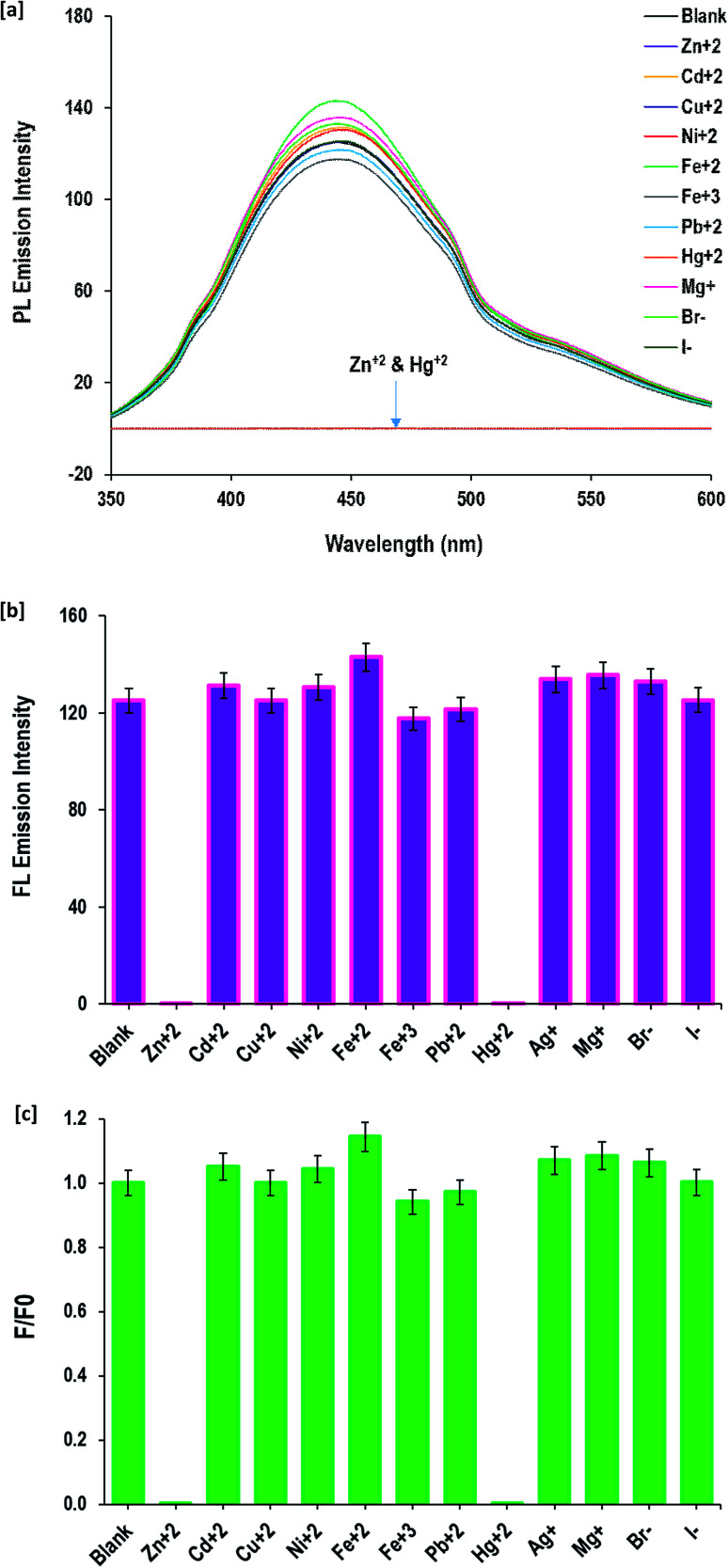
Detection of different cations and anions by the prepared CQDs; [a] fluorescence spectra and [b and c] fluorescence intensity.

One promising application of fluorescent CQDs is in probes for the detection of various metal ions to evaluate the water contamination and for bio-sensing. CQDs provide a feasible and environmentally friendly method for detection. Herein, the FL sensitivity of the as-prepared CQDs for detection of Zn(ii) and Hg(ii) ions was systematically studied. The data in Fig. 6 is the FL emission intensity of CQDs (after excitation at 340 nm) in the absence and presence of a series with metal ions [Mg^+^, Zn^2+^, Cu^2+^, Fe^2+^, Fe^3+^, Cd^2+^, Pb^2+^, Hg^+^, Ag^+^, Ni^+^, Br^−^ and I^−^]. From all of the tested metal ions, the fluorescence of CQDs was highly sensitive to Zn^2+^ and Hg^+^ ions. The fluorescence emission of CQDs was almost fully quenched in the presence of 85 mM of Zn^2+^ or Hg^+^ ions. The measurable factors of F and F0, which represented the fluorescence intensity at the same maximum excitation wavelength of 340 nm, further affirmed the highest capability of fluorescence quenching by Zn^2+^ and Hg^+^ ions.

Compared to the previous studies in the literature, the reported fluorescence of CQDs decorated with S- and/or N- was sensitive to Cu^2+^ and Fe^3+^ ions,^79,80^ while in the current work, the prepared CQDs decorated with hydroxyl groups are sensitive to Zn^2+^ and Hg^2+^. The difference in sensitivity of the fluorescence for CQDs against metal ions was attributed to the decorative groups^81^ and consequently due to the interaction of Zn^2+^ and Hg^+^ ions with hydroxyl groups as the surface decorative groups of the applied CQDs.

**Fig. 7:**
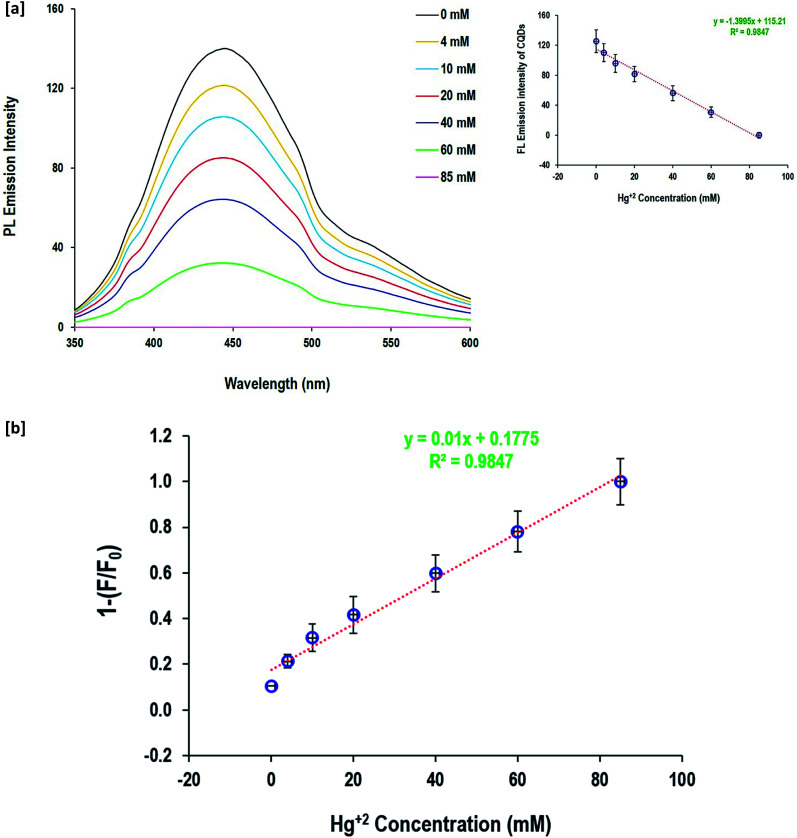
Detection of Hg^2+^ and anions by the prepared CQDs; [a] fluorescence spectra and [b] detection sensitivity.

**Fig. 8:**
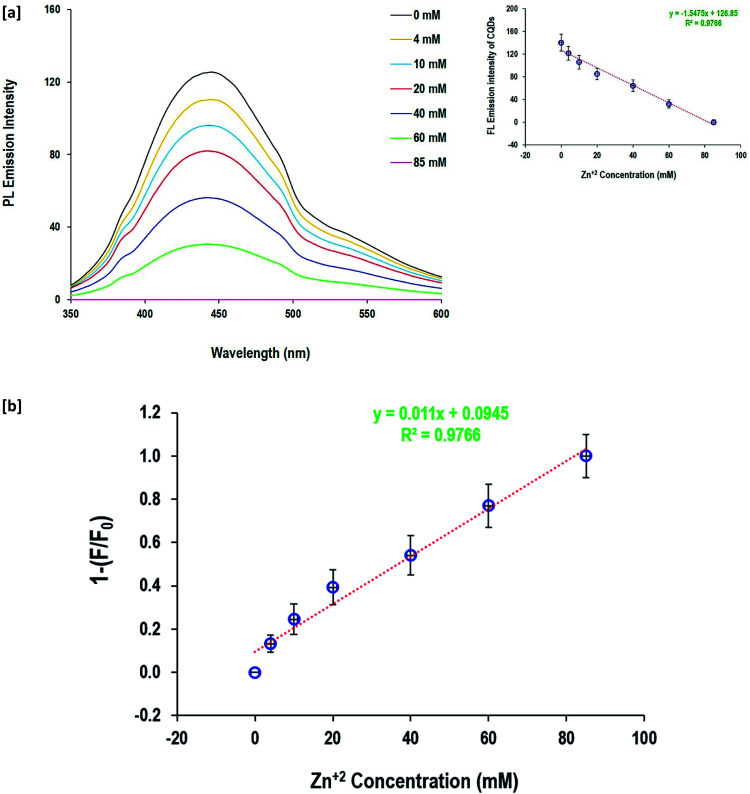
Detection of Zn^2+^ and anions by the prepared CQDs; [a] fluorescence spectra and [b] detection sensitivity.

The fluorescence quenching behavior of Zn^2+^ and Hg^+^ ions depending on their metal salt concentration was performed to evaluate the potentiality of the as-prepared CQDs in the detection of Zn^2+^ and Hg^+^ ions in aqueous solutions (Fig. 7and 8). When the Zn^2+^ and Hg^+^ ion concentration changed from 4 mM up to 85 mM, the fluorescence emission intensity of CQDs gradually diminished. The value of 1 − (*F*/*F*°) was calculated and the linear relationship with metal ion (Zn^2+^and Hg^+^) concentrations was figured out. The data showed a linear response to the gradual increase of Zn^2+^ and Hg^+^ ion concentration, as full quenching of fluorescence for CQDs was detected by 85 mM for both Zn^2+^ and Hg^+^ ions. The fluorescence of CQDs showed high sensitivity to a metal concentration of 4 mM. The calculated regression data confirmed the linear relationship between the fluorescence of CQDs and metal concentration, and the coefficient determination was quite high (*R*^2^ = 0.98). Moreover, the linear estimated equations inserted in Fig. 7 and 8 can be easily used to detect the concentration of both metal ions *via* the direct fluorescence intensity of CQDs or the calculated values of 1 − (*F*/*F*°).

According to the literature,^64^ the fluorescence quenching mechanism of the as-synthesized CQDs could be illustrated as follows; the excitation dependent fluorescence is supposed to be caused by aromatic conjugation structures, excitons of carbon, surface states, emissive traps, and free zig-zag sites. Hydroxyls as decorative groups on the surface of CQDs can form intra-molecular and intermolecular hydrogen bonds that endow the CQDs with different surface states. The interaction between heavy metals and the as-prepared fluorescent CQDs *via* the decorative hydroxyl groups result in stable complexes, causing changes in the physicochemical properties of the fluorophores, including the fluorescence intensity and anisotropy. Additionally, it provided a meaningful signal that can selectively identify the analyte with high sensitivity.^64^ Therefore, turning ON–OFF fluorescent states was mainly triggered upon mercury and zinc addition, owing to the non-radiative electron transfer from the excited state to the d-orbital of the metal ions. The formation of non-fluorescent chelating complexes between CQDs and Zn^2+^ or Hg^+^ resulted in a non-fluorescent “OFF” state.

The quenching mechanism was confirmed by the Stern–Volmer equation^82^ and the results are presented in the ESI file (Fig. S2†). The emission spectra of the fluorescent CQDs with different concentrations of Zn^2+^ and Hg^2+^ [4.0–85.0 × 10^−3^ mol] were evaluated. When the metal (Zn^2+^ and Hg^2+^) concentration ranged from 40.0–85.0 × 10^−3^ mole, the curve of *F*_0_/*F* did not fit well to the Stern–Volmer relationship. However, a good linear correlation was obtained with the metal concentrations in the range of 4.0–40.0 × 10^−3^ mol. As the metal concentration increased, the bending curve gradually ascended up, describing static and dynamic characteristics. In the case of static quenching, the interaction between CQDs and the quencher (Zn^2+^ and Hg^2+^) resulted in the production of a non-fluorescent complex. In dynamic quenching, charge or energy transfer between the excited state of CQDs and the quencher principally led to fluorescence quenching.

So, it could be summarized that the current approach presented a facile, green and cost-effective method for the synthesis of hydrophilic/fluorescent metal marker CQDs without any toxic chemicals or organic solvents. The synthesized CQDs acted as a promising fluorescent platform suitable for efficient, sensitive, rapid and selective fluorescence detection of Zn^2+^ or Hg^+^ ions, which can be further evaluated *in situ* in the environment with satisfactory and accurate results.

### Antimicrobial potency

It was reported that CQDs could successfully exhibit antimicrobial potency against various bacterial and fungal species. According to the literature,^83–90^ the mechanism of antimicrobial performance for the as-synthesized CQDs is illustrated in this section. Under visible light in aqueous medium, CQDs are capable of generating reactive oxygen species (ROS), such as singlet oxygen and hydroxyl free radicals. The produced free radicals are mainly responsible for microbial cell death. Meanwhile, ROS can adhere then penetrate the microbial cell wall to act in the induction of oxidative stress with damage to DNA and RNA, resulting in the inhibition and alteration of gene expressions. Additionally, ROS could act in mitochondrial dysfunction, lipid peroxidation, inactivation of intracellular proteins, and gradual deterioration of the cell wall, followed by apoptosis/necrosis and eventual microbial cell death.

In the presented study, the antimicrobial potency for the synthesized CQDs was estimated before and after dialysis against three different pathogenic species of +ve gram bacterial species (*B. cereus*), −ve gram bacterial species (*E. coli*) and fungal species (*C. albicans*) *via* the inhibition zone technique. Moreover, the minimal inhibitory concentration (MIC) of CQDs was evaluated. The estimated data in Table 1 strongly revealed that, against all the tested bacterial and fungal species, CQDs before and after dialysis showed excellent antimicrobial potency.

**Table 1:** Results for the antimicrobial potency for the as-synthesized CQDs *via* inhibition zone and minimum inhibitory concentration (MIC)

Sample	Inhibition zone diameter (mm per sample)			MIC (μL mL^−1^)		
	G +ve bacteria	G −ve bacteria	Fungi	G +ve bacteria	G −ve bacteria	Fungi
	*B. cereus*	*E. coli*	*C. albicans*	*B. cereus*	*E. coli*	*C. albicans*
Guar gum	0	0	0	—	—	—
CQDs before dialysis	16	15	15	500	600	600
CQDs after dialysis	21	19	17	350	450	450

**Fig. 9:**
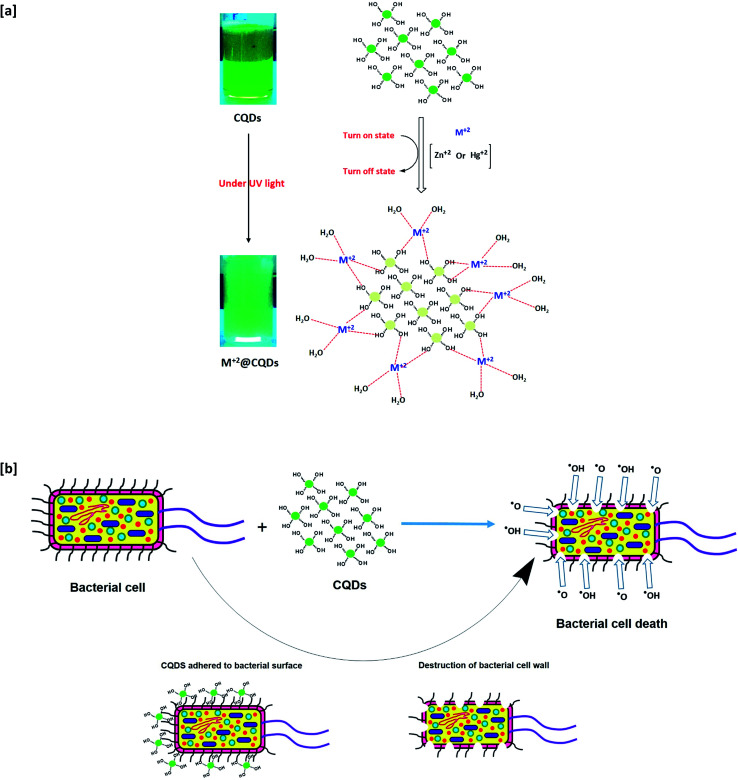
Suggested mechanisms for [a] metal detection and [b] antimicrobial action of CQDs.

While, the MIC of the as-prepared CQDs evaluated against *B. cereus*, *E. coli* and *C. albicans* were 500, 600 and 600 μL mL^−1^, and 350, 450 and 450 μL mL^−1^, for non-dialyzed and dialyzed CQDs, respectively. Of note, MIC was significantly diminished by the use of dialyzed CQDs. Meanwhile, CQDs after dialysis showed significantly enhanced antimicrobial performance owing to the effectiveness of dialysis in ultrafiltration and generation of small and size-regulated CQDs that can easily penetrate through the microbial cell membrane, causing eventual cell death. The obtained results are in agreement with the literature,^12,83,86,88,89^ while, the superiority of the as-synthesized CQDs as potent antimicrobial agents was essentially correlated to their composition of decorative hydroxyl function groups, which are mainly responsible for microbial cell death *via* the generation of reactive oxygen species (ROS), as presented in Fig. 9.

Compared with biologically active metal-based nanostructures and metal organic frameworks that were recently studied in numerous reports,^43–46^ the as-prepared CQDs exhibited significantly lower values of MIC. This reflected the superior biocidal potentiality of the synthesized CQDs, in addition to their low toxicity, biodegradability, biocompatibility and cost-effectiveness. Therefore, CQDs can be preferably applied as antimicrobial agents rather than metal-based nanostructures for biological related environmental applications.

## Conclusion

The presented study demonstrated a quite easy technique for the preparation of biocide/fluorescent metal marker CQDs as safe/potent alternatives to metallic nanostructures as probes in environmental applications. The data presented in the current approach revealed that the hydrophilic CQDs decorated with hydroxyl groups, easily synthesized by using a hydrothermal technique involving alkali-hydrolyzed guar gum, provide a green, cost-effective, facile and applicable solution. Dialysis was performed to obtain uniform-sized/highly purified CQDs. The effectiveness of the presented methodology in the synthesis of CQDs was affirmed *via* several instrumental analyses like UV-Vis spectroscopy, TEM, zetasizing, FT-IR, Raman spectroscopy and NMR spectroscopy. After 12 h hydrothermal reaction, quite small sized/regular CQDs with a size distribution of 1.5–1.7 nm were synthesized. The synthesized CQDs show stable bright-green fluorescence and were characterized by excitation dependent fluorescence properties, which show excellent potential for application in fluorescence sensing. Both the dialysis and the hydrothermal reaction duration affected the fluorescence intensity of the synthesized CQDs. The superior fluorescence sensitivity of the as-prepared CQDs was exploited in the detection of Zn^2+^ and Hg^2+^ ions. The as-synthesized CQDs could be applicable as a green fluorescent marker for the detection of zinc and mercury ions (even at 4 mM) and exhibited great performance for utilization in high-end applications. The fluorescence quenching mechanism was studied by the Stern–Volmer equation. Additionally, the antimicrobial potency of the synthesized CQDs was estimated and the data affirmed their excellence biocidal performance against all the tested bacterial and fungal species.

The presented approach could be described as an alternative simple technique without use of any toxic chemicals or organic solvents and able to achieve the large scale synthesis of hydrophilic CQDs as biocide/fluorescent markers. Moreover, the current study will open new ways for the simple preparation of highly effective CQDs from natural biopolymers to be used as probes for various environmental and biological related purposes instead of metal-based nanomaterials.

## Conflicts of interest

The authors declare that they have no conflict of interest

## Supplementary Material

RA-010-D0RA06383E-s001
