# Supplementary material for: Environmentally exploitable biocide/fluorescent metal marker carbon quantum dots
Source: RSC Adv. 2020 Nov 26;10(70):42916–29. doi: 10.1039/d0ra06383e (PMC9058413; doi:10.1039/d0ra06383e)
Supplement: RA-010-D0RA06383E-s001 [file RA-010-D0RA06383E-s001.pdf]

Electronic Supplementary Material (ESI) for RSC Advances.
This journal is © The Royal Society of Chemistry 2020

## Supplementary data

**Figure S1:** The reproducibility results for the prepared CQDs (12 h, after dialysis); [a] size distribution and [b] Fluorescence spectra.

**[a]**

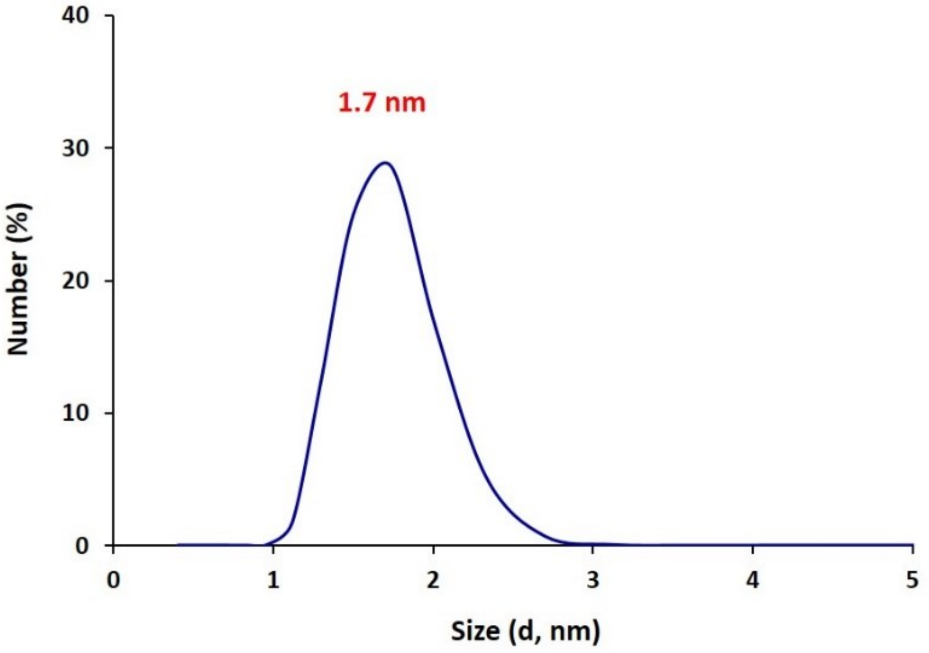


**[b]**

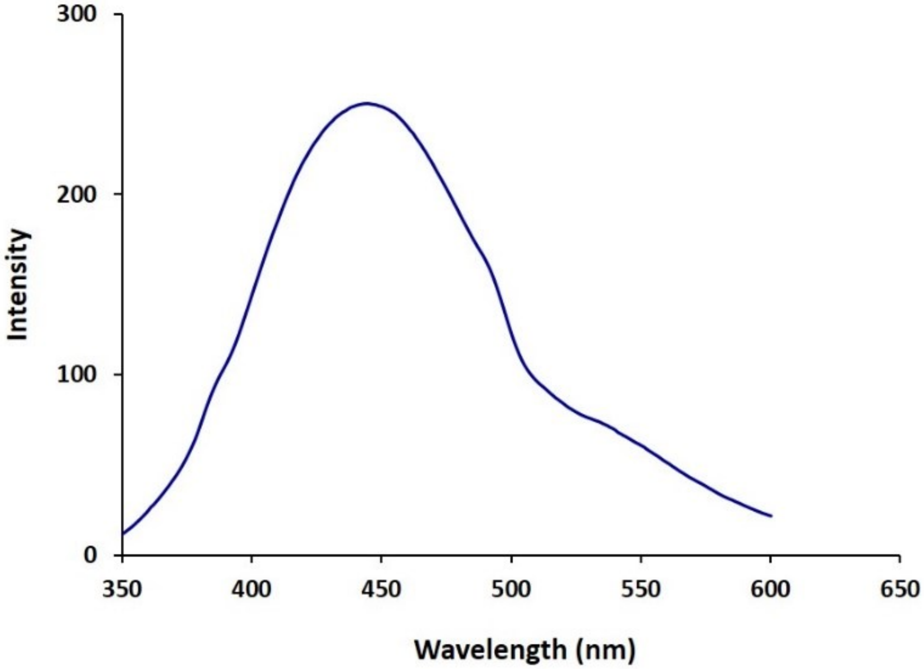

**Figure S2:** Stern–Volmer model for the fluorescence quenching of CQDs by metal ions ($Zn^{+2}$ & $Hg^{+2}$), the inset figures presented the only linear relationship.

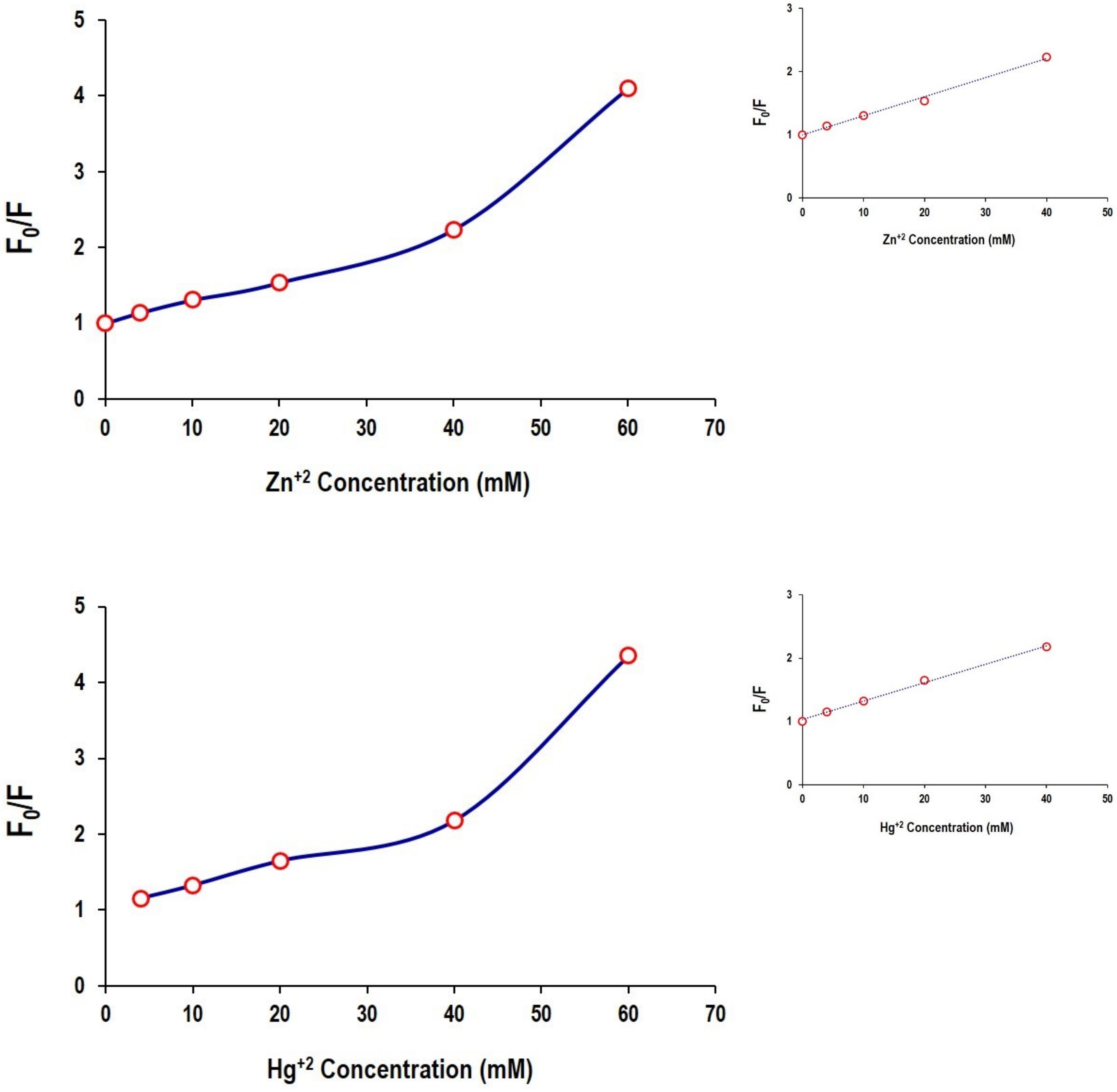

**Figure S3:** Photographic images for antimicrobial

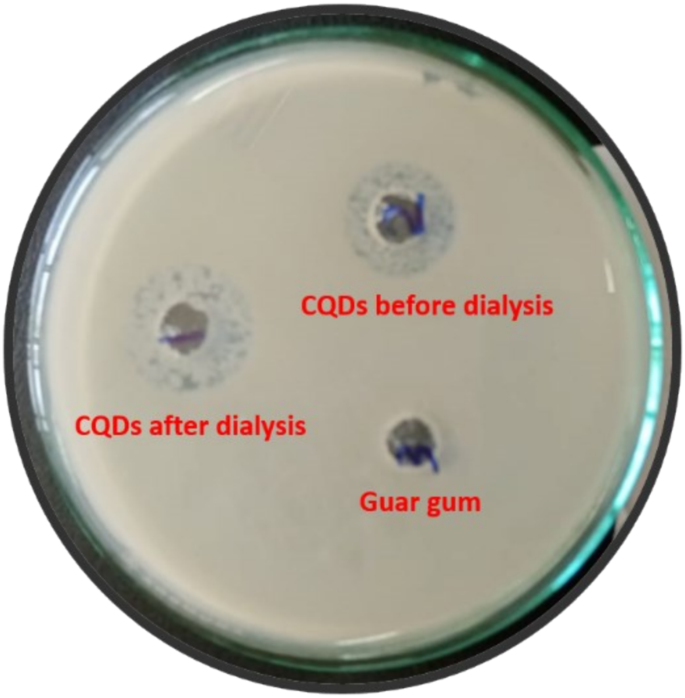


**B. Cereus**

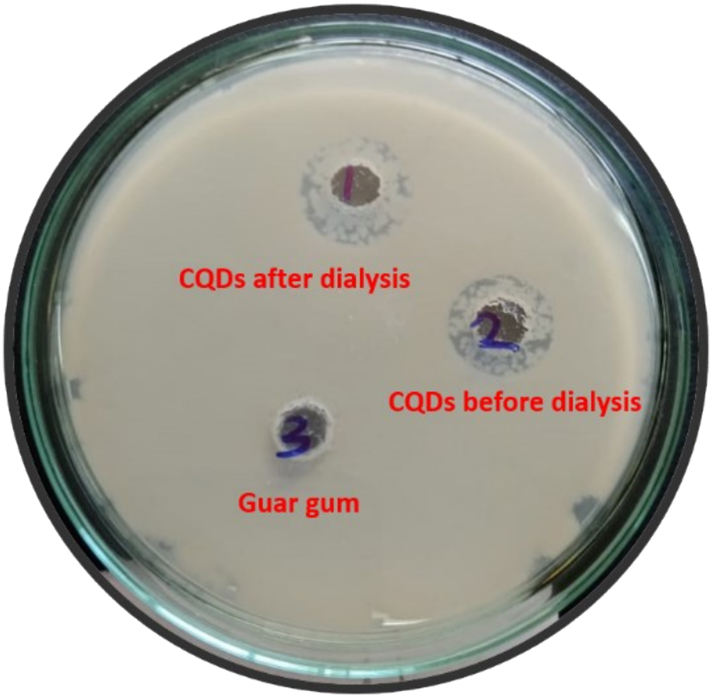


**C. Albicans**
